# IDO Expression in Cancer: Different Compartment, Different Functionality?

**DOI:** 10.3389/fimmu.2020.531491

**Published:** 2020-09-24

**Authors:** Annabel Meireson, Michael Devos, Lieve Brochez

**Affiliations:** ^1^Department of Dermatology, Ghent University Hospital, Ghent, Belgium; ^2^Cancer Research Institute Ghent, Ghent, Belgium

**Keywords:** IDO, kynurenine, tryptophan, cancer, indoleamine (2,3)-dioxygenase, tumor immunity

## Abstract

Indoleamine 2,3-dioxygenase 1 (IDO1) is a cytosolic haem-containing enzyme involved in the degradation of tryptophan to kynurenine. Although initially thought to be solely implicated in the modulation of innate immune responses during infection, subsequent discoveries demonstrated IDO1 as a mechanism of acquired immune tolerance. In cancer, IDO1 expression/activity has been observed in tumor cells as well as in the tumor-surrounding stroma, which is composed of endothelial cells, immune cells, fibroblasts, and mesenchymal cells. IDO1 expression/activity has also been reported in the peripheral blood. This manuscript reviews available data on IDO1 expression, mechanisms of its induction, and its function in cancer for each of these compartments. In-depth study of the biological function of IDO1 according to the expressing (tumor) cell can help to understand if and when IDO1 inhibition can play a role in cancer therapy.

## Introduction

Indoleamine 2, 3-dioxygenase 1 (IDO1, hereafter referred to as IDO) is a 403 amino acid cytosolic haem-containing enzyme involved in the first, rate-limiting step of the tryptophan (Trp) metabolism to kynurenine (Kyn) (1, 2). Trp is an essential amino acid for which both neuropsychological as well as immunological functions have been described. Despite their shared function in Trp degradation, the IDO2 isoform and tryptophan 2, 3-dioxygenase (TDO2) have distinct inducers and patterns of tissue expression (3, 4).

*IDO* (human chromosome 8p22) is recognized as an interferon (IFN)-inducible gene. Indeed, the promoter region of *IDO* consists of several IFN-stimulated response elements (ISREs) and gamma activation sequences (GAS), permitting a controlled and context-dependent transcriptional process (2, 5, 6).

**Figure 1:**
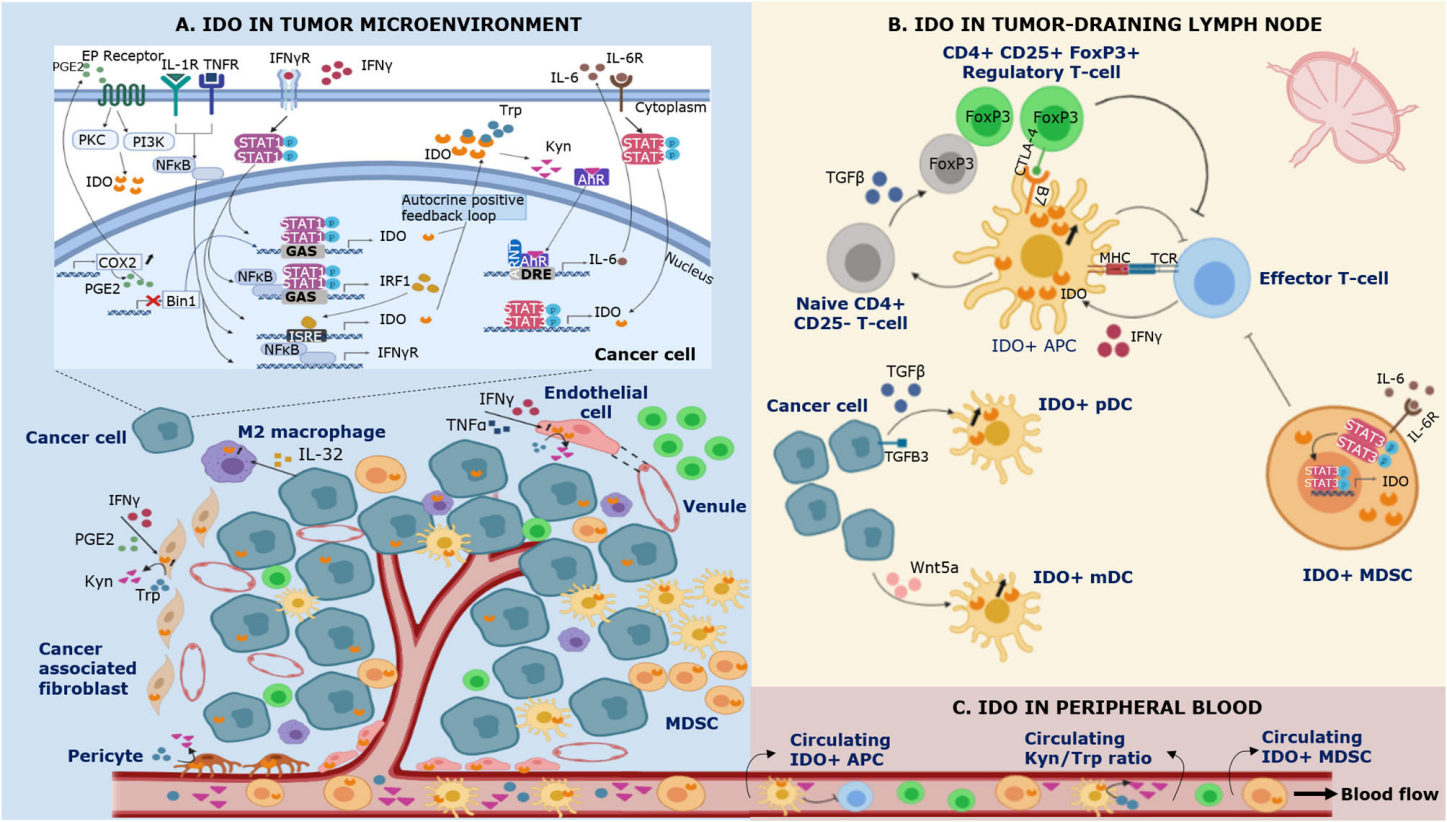
Schematic representation of IDO expression in different compartments of the immune system during cancer. IDO is expressed by multiple cell types in the tumor microenvironment **(A)**, the tumor-draining lymph node **(B)** and the peripheral blood **(C)**. **(A)** Bin1 attenuation results in STAT1- and NFκB-dependent constitutive expression of IDO in cancer cells. In addition, COX2 overexpression facilitates constitutive IDO expression via PGE2-mediated activation of the PKC/PI3K pathways. IFNγ is recognized as a highly potent inducer of IDO expression. Binding of IFNγ to its receptor (IFNγR) leads to (i) tyrosine phosphorylation of STAT-1, triggering its dimerization and binding to the GAS sequence in *IDO1* and (ii) NF-κB and STAT-1 dependent synthesis of IFNγ-regulated factor 1 (IRF1), which binds to the ISRE sequences in *IDO1*. Tumor IDO expression activates the cytosolic transcription factor aryl hydrocarbon receptor (AhR) by kynurenine (Kyn), stimulating an autocrine positive feedback loop via IL-6 dependent STAT-3 signaling which maintains IDO expression. In addition to IFNγ, IDO expression can be induced by other proinflammatory cytokines such as tumor necrosis factor α (TNFα) and IL-1 who enhance the expression of IFNγR on cancer cells. IFNγ and TNFα can also induce IDO expression in endothelial cells of venules in the tumor microenvironment. In the tumor-surrounding stroma, IDO is expressed by cancer associated-fibroblasts, pericytes, and infiltrating immune cells. **(B)** Regulatory T-cells (Tregs) induce IDO expression by antigen-presenting cells (APCs) via CTLA-4/B7 ligation in the tumor-draining lymph node. In addition, cancer cells are involved in the upregulation of IDO expression in plasmacytoid dendritic cells (pDCs) by shedding of the extracellular domain of the type III TGF-B receptor (sTGFBR3). IDO expression in myeloid DCs (mDCs) can be induced by cancer cell-secreted Wnt5a, which triggers binding of β-catenin to its responsive elements. IDO^+^ APCs inhibit T-cell responses and polarize naïve CD4^+^ T-cell differentiation toward the phenotype of suppressive Tregs via TGFβ-mediated FoxP3 upregulation. Myeloid derived suppressor cells (MDSCs) upregulate IDO via IL-6 triggered STAT-3 activation. **(C)** IDO^+^ APCs and IDO^+^ MDSCs infiltrate the tumor microenvironment and the peripheral blood, contributing to local and systemic immune escape.

Although initially thought to be solely implicated in the modulation of innate immune responses in parasitic/viral conditions (7–9), subsequent discoveries demonstrated IDO to be a mechanism of acquired immune tolerance (4). In cancer, IDO expression has not only been documented in tumor cells but also in endothelial cells, fibroblasts and immune cells infiltrating the tumor microenvironment (Figure 1). In addition to the local tumor microenvironment, IDO expression was detected in peripheral blood mononuclear cells (PBMCs) in blood samples of cancer patients. Although IDO expression has been reported in these different compartments, the exact mechanisms for its distinct expression patterns and their functions are far from completely understood. In view of the complex interplay between malignant cells and their microenvironment, understanding IDO activation and its particular function in the different compartments may be of the outmost importance. This review summarizes the available scientific data.

## IDO In the Tumor Microenvironment

IDO expression in the tumor microenvironment has been described in tumor cells, immune cells, endothelial cells, and stromal fibroblasts (Table 1).

**Table 1:** Regulatory mechanisms and functions of IDO expressing cells in the tumor microenvironment.

		**Human studies**	**Animal studies**
Tumor cells	Regulatory mechanisms	- **Constitutive expression** (10, 11) dependent on COX2 and PGE2 (12) - **Induction by type I,II IFNs** (13), mediated by STAT-1 and NF-κB signaling (2, 5, 6, 14, 15) - Expression inversely correlated with Bin expression (16, 17) - Synergism between IFNγ and TNFα/IL1 on expression (18–20) - **Autocrine positive feedback loop** through Kyn-AhR-IL6-STAT3 (11)	- STAT-1- and NF-κB-dependent expression as a consequence of loss of Bin (21) - LPS induces systemic activity dependent on TNF (22)
	Functions	- Associated with **increased intratumoral Treg** infiltration and **impaired cytotoxic T-cell function** (23–30) - Conversion of CD4^+^CD25^−^Treg into CD4^+^CD25^+^ cells (31) - Prevents degranulation of CD8^+^ and γδ T-cells (32, 33) - Associated with **MDSC infiltration** (34) - Promotes **proliferation of HUVEC cells** (35) - Associated with metastasis (35–42) - Drives **dormancy** of tumor repopulating cells (11, 43) - Correlates with PD-1 and PD-L1 expression (29, 30, 44–46)	- Trp depletion/catabolites drive TCR ζ-chain downregulation in CD8^+^ cells and induce FoxP3 in CD4^+^ cells (47) - Drives IL-6 dependent MDSC-mediated immune escape (36) - Associated with enhanced VEGF-C expression and lymphangiogenesis (48–50) - Promotes tumor vascularization (36) - Drives dormancy of tumor repopulating cells (43) - Drives tumorigenesis through NAD^+^-depletion induced DNA damage (51)
Immune cells	Regulatory mechanisms	- Induction by PGE2 and activated by TNFα/TLR signaling in moDC (52, 53) - Induction by synergystic combination of IFNγ and CD40L in monocyte-derived macrophages (54) - Induction by IL-32γ depending on NF-κB and STAT-3 in macrophages (55) - Induction in MDSCs requires phosphorylation of STAT-3, but not STAT-1 (39) and non-canonical NF-κB (56)	- Expression in splenic DCs induced by CpGs and dependent on IFN type I signaling (57) - Expression in DCs induced by Tregs through CTLA-4/B7 (58) - Expression in pDCs induced by CpG, TGFβ, CD200 (59–61) - Expression in mDCs induced by Wnt5a (62, 63) and IFNγ in an IRF8-dependent way, functionally active in cDC1 (64, 65)
	Functions	- **DCs decrease antigen uptake and downregulate CD40/CD80** under low Trp conditions (66) - Expression in moDCs induces regulatory activity in T-cells (67, 68) - Expression is associated with distinct profile of cytokines and surface markers in moDCs (69, 70) - Mediates IFNγ-induced differentiation of monocytes into **M2-macrophages** (71–74) - Expression in macrophages halts cell cycle progression in T-cells (54, 55) - Expressing MDSCs associated with **FoxP3**^**+**^ **Tregs and impaired CD8**^**+**^ **T-cell function** (39, 75, 76)	- Expression in pDCs suppresses T-cell responses in TDLN (77) and activates Tregs in tumor microenvironment and TDLN (60, 61, 78) - Expression in DCs and MDSCs implicated in anti-PD-1 resistance (79) - Expression in MDSCs impairs AMPK and mTOR function (80)
Endothelial cells	Regulatory mechanisms	Induction and synergism by IFNγ (81, 82) and TNF (81)	- Induced by IFNγ through non-canonical NF-κB activation (83) - Induced by agonistic CD40 mAb through IFNγ secretion by CD8^+^ T-cells (84)
	Functions	- Expression in CD31^+^ HEV in peritumoral stroma: sentinel LN and metastatic tissue associated with **reduced CD8**^**+**^ **T-cells** and **increased FoxP3**^**+**^ **Tregs** (85) - Expression in sentinel LN associated with enhanced IDO expression in peripheral blood (86) - Expression associated with microsatellite instability in CRC (87) - Expression associated with responsiveness to anti-PD-1 (88, 89) - Drives tumor dormancy through Trp depletion-induced TSLP expression/secretion (90) - Expression in LECs **impairs CD4**^**+**^ **T-cell proliferation** (91)	
Stromal cells	Regulatory mechanisms	- Induction in CAFs (92, 93), mesenchymal stem cells (94–96) and pericytes (82) by IFNγ - Expression in CAFS mediated by COX2/PGE2 (97)	
	Functions	- Expression in CAFs **suppresses NK cell activity** (98, 99) - Expression in dermal fibroblasts induces **apoptosis in T-cells, B cells and monocytes** (100) - Expression in mesenchymal stem cells involved in **inhibition of T-cell function** (96) and conversion of monocytes into **M2-macrophages** (101) - Expression in pericytes **negatively regulates T-cell proliferation** (102)	Expression in CAFs (103) and mesenchymal stem cells (104) involved in Treg activation

### Tumor Cells

The majority of literature reports on IDO positivity in neoplastic cells. Strong expression in tumor tissue is identified as an independent negative prognostic factor in multiple cancers (4, 35, 87, 105, 106). It is well-documented that tumoral IDO expression is associated with tumor-infiltrating forkhead box P3 positive regulatory T-cells (FoxP3^+^ Tregs) and IDO-expressing mononuclear cells, while a negative association with CD8^+^ cytotoxic T-cells in the primary tumor and metastatic tissue has been reported (23–30, 107). These observations in human samples are consistent with earlier mechanistic studies elucidating the involvement of IDO in impairing cytotoxic effector T-cell function/proliferation via downregulation of the T-cell receptor ζ chain as well as its stimulatory role in enhancing Treg generation (31, 47, 54, 108, 109). In diffuse-type gastric cancer, elevated tumoral IDO expression correlated with decreased expression of CD107a and granzyme B in tumor-infiltrating CD8^+^ T-cells, reflecting T-cell dysfunction (32). IDO expressed by pancreatic ductal adenocarcinoma cells was observed to support immune escape of cancer cells by impairing cytotoxicity and degranulation of γδ T-cells (33). In addition to FoxP3, tumoral IDO expression has been evidenced to be strongly correlated with other immunosuppressive molecules such as programmed cell death protein 1 (PD-1) and its ligand PD-L1 (29, 30, 44–46).

The extent of tumoral IDO expression has been investigated in the context of deficiencies in the DNA mismatch repair system. The microsatellite instable (MSI-H) subgroup of colorectal cancer is characterized by a strong infiltration of activated cytotoxic T-lymphocytes, which is a positive prognostic factor (110–113). Despite this highly inflamed environment, MSI-H tumors persist in such hostile climate due to overexpression of immune checkpoint molecules as cytotoxic T-lymphocyte-associated protein 4 (CTLA-4), lymphocyte-activation gene 3 (LAG-3), PD-1/PD-L1, and IDO, hampering an efficient anti-tumor T-cell response (87, 114, 115). It is hypothesized that the active tumor microenvironment is stimulated by an increased neoantigen load in MSI-H tumors, which is counterbalanced by the upregulation of immune checkpoints, such as IDO, as a negative feedback mechanism. This immunosuppressive climate mediates evasion of the tumor from the host immune system.

Besides the suppression of anti-tumor immune responses, tumoral IDO is involved in tumor vascularization. IDO-deficiency was observed to significantly decrease pulmonary vascular density in lung cancer mouse models, predominantly reducing small to medium size vessels, unaltering large vessels (36). In breast cancer, a cancer cell line (MCF-7) with strong IDO expression promoted proliferation of human umbilical vein endothelial cells (35). In murine metastasized melanoma lymph nodes, tumoral IDO expression was associated with enhanced expression of VEGF-C, an inducer of lymphangiogenesis, previously linked to the occurrence of regional lymph node metastasis (48–50). This observation suggests tumoral IDO expression is involved in the expansion of lymphatic vessels. Furthermore, tumoral IDO expression has been proposed to stimulate the metastasic process. An association between strong IDO expression at the primary tumor and development of lymph node and/or metachronous metastases is described in various malignancies (36–42). Studies detecting IDO in the primary tumor and the corresponding lymph node and metastatic tissue reported a highly consistent expression pattern of IDO throughout the disease course (44, 87). Altogether, these data define tumoral IDO as a modulator that bridges inflammation, vascularization, and immune escape to promote primary and metastatic tumor outgrowth.

Although it is widely accepted that tumor cells are capable of expressing IDO, the critical signals directing its expression and activity are only partially revealed. There are indications for constitutive/intrinsic as well as induced/extrinsic tumor IDO expression. Constitutive expression of *IDO* mRNA in the absence of any IFNγ exposure has been demonstrated in several cancer cell lines (10). This study also investigated *in vivo* IDO expression in multiple malignancies and normal cells in the stroma were observed to be IDO-negative in contrast to the tumor cells. The authors concluded that this tumoral IDO expression could not be the result of IFNγ exposure, as this would have induced IDO in the surrounding stroma too. Another study in ovarian and adeno-squamous lung cancer cell lines demonstrated that cancer cells expressed *IDO1* mRNA and constitutively released Kyn into the supernatant (11).

Loss of the tumor suppressor Bridging Integrator 1 (Bin1) and overexpression of cyclooxygenase-2 (COX2) are both linked to intrinsic upregulation of IDO. Bin1 loss in a knockout mouse model was associated with elevated STAT1- and NFκB-dependent expression of IDO, driving tumor immune escape (21). This is supported *in vivo* by the observation that tumor expression of Bin1 is inversely correlated with IDO expression in esophageal squamous cell cancer and lung cancer (16, 17). COX2 has been implicated in the pathogenesis of several cancers, in particular colorectal cancer, where it impacts oncogenic signaling, invasion and metastasis, survival and angiogenesis (116–118). In a series of tumor cell lines, it was demonstrated that constitutive IDO expression depends on COX2 and prostaglandin E2 (PGE2), which upon autocrine signaling through the EP receptor activates IDO transcription via the PKC and PI3K pathways. Oncogenic mutations were identified in the signaling pathways involved in this autocrine loop, favoring constitutive IDO expression (12).

Type I and especially type II IFNs are known to be potent IDO-inducers (13). As tumor-infiltrating lymphocytes (TILs) are a predominant source of IFNγ, they might upregulate IDO as a negative feedback signal, hereby potentially contributing to tumor immune escape. This is in line with the observation that human hepatoma cell lines express IDO once T-lymphocytes and monocytes are added, subsequently upregulating IFNγ in the co-culture (18). IFNγ-dependent induction of tumoral IDO expression has been extensively analyzed in various malignancies (38, 88, 119, 120). IFNγ-mediated signal transduction leads to (i) tyrosine phosphorylation of STAT-1, triggering its dimerization and binding to the GAS sequence in *IDO* and (ii) NFκB- and STAT-1-dependent synthesis of IFNγ-regulated factor 1 (IRF1), which binds to the ISRE sequences in *IDO*. Combined STAT-1 and IRF-1 binding to GAS and ISRE sequences in the *IDO1* gene promoter is necessary for maximal IFNγ-mediated induction of IDO transcription (2, 5, 6, 14, 15). Tumoral IDO expression was suggested to stimulate an autocrine positive feedback loop via the activation of the cytosolic transcription factor aryl hydrocarbon receptor (AhR) by Kyn. AhR activation subsequently upregulates IL-6, which mediates STAT-3 signaling driving IDO expression (11). In addition, the IDO-Kyn-AhR pathway has been evidenced to drive dormancy in tumor repopulating cells (TRCs), a highly tumorigenic subpopulation of cancer cells involved in the initiation and progression of tumorigenesis. When TRCs were stimulated *in vitro* with IFNγ, phosphorylated STAT-1 rapidly upregulated IDO expression, subsequently elevating Kyn levels and activating AhR. This pathway triggers G0/G1 cell cycle arrest by p27 and TRC dormancy (43).

IFNγ-mediated IDO induction can be potentiated by other proinflammatory cytokines, such as tumor necrosis factor α (TNFα) (18, 19), IL-1 (20), lipopolysaccharide (LPS) (7, 22), CpG oligodoxynucleotides (57) and PGE2 (52). The combination of these inflammatory stimuli results in synergistic enhancement of *IDO* transcription. For instance, IL-1 and TNFα enhance the expression of IFNγ receptors (IFNγRs) via the transcription factor NFκB, lowering the threshold for IFNγ-directed IDO upregulation (121). In addition to IFNγ, TNFα synergistically induces IDO expression by increasing both STAT-1 activation and NFκB -dependent IRF-1 expression (19).

Immunohistochemical analysis of biopsies of melanoma metastases detected IDO, PD-L1, and FoxP3 in CD8^+^ T-cell inflamed regions (29). In contrast, non-T-cell inflamed melanomas lacked these factors, suggesting that immune suppression might not be a property of tumor cells but rather an immune-intrinsic negative feedback process that follows the infiltration of activated CD8^+^ T-cells. These data indicate that IFNγ produced by CD8^+^ T-cells is a requisite factor for PD-L1 and IDO expression in melanoma metastatic tissue (29). Such T-cell inflamed–also termed immunologically “hot” –tumors have been associated with higher response rates to anti-PD-1 immunotherapy (122). This is in contrast to T-cell non-inflamed tumors–also referred to as “cold” tumors–which might constitute a group of tumors expressing IDO in absence of any inflammation and T-cell infiltration, representing a state of intrinsic immune resistance.

CD8^+^ T-cell-mediated IDO expression via IFNγ in immunologically “hot” tumors could be one of the explanations why studies in breast cancer (123, 124), gastric adenocarcinoma (125), hepatocellular (126), pancreatic cancer (127), adenosquamous lung carcinoma (128), and prostate cancer (129) observed a positive prognostic effect for tumoral IDO expression. Another explanation–apart from technicalities such as the use of different antibody clones that could result in distinct staining patterns–could be Trp shortage caused by IDO activity. Trp is the only endogenous precursor for *de novo* biosynthesis of nicotinamide adenine dinucleotide (NAD^+^), which is a co-enzyme of redox reactions for adenosine triphosphate (ATP) production. Enhanced IDO activity results in downregulated Trp and NAD^+^ levels, the latter being a vital co-factor in energy production, DNA synthesis, and cellular homeostasis. NAD^+^ depletion-induced DNA damage has been evidenced to play a role in liver tumorigenesis (51, 130).

### Immune Cells

The most extensively studied IDO-expressing immune cell types in the tumor microenvironment are antigen presenting cells (APCs) and myeloid derived suppressor cells (MDSCs).

Immunohistochemical analysis of the local tumor microenvironment identified IDO expression in human dendritic cells (DCs) in melanoma (131), breast cancer (45), squamous cell carcinoma (132), Hodgkin lymphoma (71), and esophageal cancer (133). It is well-known that DCs acquire a strong tolerogenic capacity when cultivated under low Trp conditions as they decrease antigen uptake and downregulate the expression of the costimulatory molecules CD40 and CD80 (66). Munn et al. (77) detected IDO expression in murine plasmacytoid dendritic cell (pDCs) subsets upon CTLA4-Ig exposure. Although human CD11c^−^ CD123^+^ pDCs constitute a minor part of the tumor infiltrate, *in vitro* experiments in murine tumor draining lymph nodes (TDLNs) demonstrated that pDCs potently suppress CD8^+^ T-cell responses to (i) antigens presented by the pDCs themselves, but also to (ii) third-party antigens presented by non-suppressive APCs (78). Notably, all of the T-cells achieved anergy when cultivated *in vitro* with a low quantity of IDO-expressing DCs, suggesting that *in vivo* levels (estimated at 0.5% of all TDLN cells) are sufficient to direct the entire TDLN toward a tolerogenic climate. The immunosuppressive effects of IDO^+^ pDCs are elicited by inhibitory effects on CD8^+^ T-cell responses, but also by GCN2-dependent activation of mature CD4^+^ CD25^+^ Tregs. *In vitro* CTLA-4 blockade significantly inhibited IDO-induced activation of Tregs in co-cultures, underlining the essential role of CTLA-4 in this pathway. Importantly, Tregs can trigger upregulation of IDO expression in DCs via CTLA-4 ligation with B7 receptor molecules on DCs (58). Fully activated Tregs reciprocally upregulate PD-L1 and PD-L2 expression on target DCs, suggesting IDO-induced Treg activation proceeds via a self-amplifying loop. Reverse signaling via other ligand-receptor pathways than CTLA-4/B7 interaction to induce IDO expression on DCs such as GITR, ICOS and CD200 has also been reported (59, 134, 135). In addition to the described rapid and potent mechanism of activating mature Tregs, IDO^+^ pDCs also upregulate TGFβ-mediated FoxP3 expression in naïve CD4^+^ CD25^−^ cells, hereby polarizing CD4^+^ T-cell differentiation toward the phenotype of suppressive Tregs. IL-6 production is simultaneously blocked in these naïve CD4^+^ CD25^−^ cells which prevents their conversion into Th17-like effector T-cells (47, 60). Intriguingly, tumor cells are involved in the upregulation of IDO expression in pDCs by shedding of the extracellular domain of the type III TGF-B receptor (sTGFBR3). A decrease in tumor-associated TGFBR3 expression increased TGFβ-dependent upregulation of IDO in pDCs within the primary tumor and TDLN of murine models of breast cancer and melanoma (61).

Myeloid conventional DCs (mDCs, characterized by CD11c^+^ CD123^−^) are also documented to express IDO. Despite the fact that both murine CD8α^+^ and CD8α^−^ dendritic cells (resp. cDC1 and cDC2) express IDO upon *in vitro* stimulation with IFNγ, only IDO expression on cDC1s seems to be functionally active as evidenced by the Kyn concentration in the supernatant. Addition of IFNγ-stimulated cDC1s to Th1 cells caused up to 40% of Th1 cells to undergo apoptosis. In case of cDC2s, the proportion of Th1 cells undergoing apoptosis was equally low when co-cultured with unstimulated or IFNγ-stimulated cDC2s (64, 65). Besides IFNγ-mediated upregulation of IDO expression in mDCs, tumor cells promote mDC tolerization in the tumor microenvironment via paracrine Wnt-mediated signaling. Wnt5a secreted by melanoma cells activates β-catenin in DCs which upon nuclear translocation binds the TCF/LEF1 transcription factor responsive elements subsequently inducing IDO expression in an IFNγ-independent manner (62, 63). IDO expression in murine lung cancer models was uniquely observed in CD11b^+^ CD11c^−^ DCs, which were predominantly CD8α^−^ (79). IDO expression has also been detected in a rare murine splenic cell type having phenotypic attributes of cDC1s (CD8α^+^, CD80/CD86, MHCII) combined with expression of markers of the B-cell lineage (CD19^+^, Pax5 and surface Ig) (136).

In addition to DCs differentiating from common dendritic progenitor cells (pDCs and mDCs), human monocyte-derived DCs (moDCs) are identified as IDO-competent APCs. Immunomodulatory properties of IDO^+^ moDCs are identical to those described for pDCs and mDCs, including stimulation of Treg differentiation from naïve CD4^+^ CD25^−^ cells and suppression of T-cell responses (53, 67, 68). IDO expression seems to be dependent on the maturity status of moDCs and is limited to CD83^+^ moDCs. Compared to IDO^−^ moDCs, IDO^+^ moDCs release a different pattern of cytokines (less IL-6 & IL-10, more IL-1β & IL-15) and upregulate surface markers as CD80, CD86, PD-L1, and PD-L2 (69). The different cytokine expression profile suggests altered functionality in IDO^+^ moDCs. Remarkably, direct cell-contact between immature moDCs and mast cells has been observed to upregulate IDO in moDCs. This depends on interaction between PD-1, expressed by tissue resident mast cells, and PD-L1/PD-L2 on moDCs (70).

A role for IDO in the IFNγ-mediated differentiation of monocytes into M2-type macrophages has also been proposed (72, 73). M2-macrophages are associated with tumor progression in prostate, colon, breast cancer, gastric and ovarian cancer (137–144). *In vitro* stimulation of monocytes with IFNγ increased the M2/M1 ratio, while silencing of IDO in monocytes resulted in upregulation of pro-inflammatory M1-macrophages (74). In melanoma, macrophages constituted the predominant source of IDO expression in brain metastases (145). In agreement with these observations, IDO expression was detected in CD163^+^ (M2-type) macrophages infiltrating the tumor microenvironment in Hodgkin lymphoma, and associated with shortened survival in these patients (71). A negative prognostic effect was confirmed in an independent Hodgkin lymphoma cohort, and the proportion of macrophages expressing both IDO and PD-L1 correlated with IFNγ gene expression (146). *In vitro* stimulation of human monocyte-derived macrophages showed that the early T-cell activation marker CD40L synergized with IFNγ for IDO upregulation (54). IDO-expressing macrophages interfered with T-cell activation, halting cell-cycle progression in the G1-phase. In multiple myeloma patients, tumor cells were described to secrete IL-32, triggering phosphorylation of STAT-3 and nuclear translocation of NFkB, subsequently inducing IDO expression in macrophages (55). Moreover, IDO^+^ IL-32-educated macrophages suppressed proliferation of CD4^+^ T-cells when co-cultured *in vitro*.

Strong expression of IDO by tumor cells associates with a higher level of tumor-infiltrating MDSCs in melanoma (34). MDSCs are myeloid cells with potent suppressive activities against effector lymphocytes in tumor immunology (147). IL-6 was found to be critical as an effector cytokine of IDO-driven MDSC activity and subsequent metastasis in lung cancer (36). MDSCs are also capable of expressing IDO, promoting tumor growth, and T-cell inhibition. The frequency of IDO^+^ MDSCs was positively associated with the amount of FoxP3^+^ Tregs and had a negative impact on patient outcome in breast cancer patients receiving neoadjuvant chemotherapy (75). In the tumor microenvironment of murine lung cancer models a subgroup of monocytic (Gr1^int^ CD11b^+^) MDSCs were defined as the main source of IDO expression (79). IDO^+^ MDSCs in a lung cancer mouse model were evidenced to impair AMPK and mTOR function, which are metabolic regulators in energy homeostasis during cellular stress (80). Because of reduced signaling of these regulators, tumor-residing CD8^+^ T-cells upregulate checkpoint molecules, such as PD-1, CTLA-4, LAG-3, and TIM-3, reflecting the exhausted state of these cells. The molecular mechanisms underlying aberrant expression of IDO in MDSCs remain partially unclear. As described above, IFNγ is the most potent inducer of IDO expression in DCs and macrophages. IFNγ-triggered IDO expression mainly occurs trough the STAT-1 pathway (5, 57, 148). However, in human breast cancer or hematological cancer no changed IFNγ expression or STAT-1 signaling in MDSCs could be observed (39, 76). IDO was upregulated in breast cancer-derived MDSCs via IL-6-triggered STAT-3 activation, which activated the non-canonical NFκB pathway resulting in enhanced transcriptional activity of the IDO promoter (56).

### Endothelial Cells

Physiological expression of IDO in endothelial cells (ECs) is limited, but has been extensively demonstrated in vessels of the villous chorion and in the spiral arteries of the decidua in humans during pregnancy (149). During the course of pregnancy endothelial IDO expression extends from the subtrophoblastic capillaries to larger vessels in the villi and the chorionic plate (150). Gestational age is associated with an increased Kyn/Trp ratio in the placenta, reflecting enhanced IDO activity. Endothelial IDO expression during pregnancy has been implicated in various important functions such as immune tolerance, antimicrobial protection, and optimization of placental perfusion (151–155). Antimicrobial as well as immunoregulatory properties have been designated to IDO-positive ECs. Stimulation of human brain microvascular ECs with IFNγ restricted growth/replication of viruses, bacteria, and parasites (153, 156, 157). In addition to these antimicrobial effects, IDO-mediated degradation of Trp in brain microvascular ECs is responsible for a significant reduction of T-lymphocyte proliferation. This is in line with observations made in IDO-transfected ECs, which failed to stimulate allogeneic T-cell responses while anergy was induced in allospecific T-cells (81). A role for IDO-expressing ECs in the regulation of blood pressure has been observed, as IDO activity in murine ECs—measured by Kyn and Trp concentrations in plasma—resulted in arterial vessel relaxation through involvement of adenylate and soluble guanylate cyclase pathways (158). Furthermore, increased plasma Kyn/Trp has been associated with endothelial dysfunction and dysregulated immune responses during human sepsis (159, 160). Intriguingly, a recent study reported that patients with microvascular endothelial dysfunction had a >2-fold increased risk of developing solid-tumor cancer over a median follow-up period of 6 years (161).

Endothelial IDO expression has been described in several malignancies (71, 85, 87, 88, 90, 162, 163). In melanoma, IDO expression was prominent in CD31^+^ high endothelial venules (HEV) in the stroma surrounding the tumor (85). Endothelial IDO expression in the peritumoral stroma of the primary tumor was consistent with expression in the corresponding sentinel node. Notably, IDO positivity in ECs persisted in metastatic melanoma tissue developing at a median time of 3.4 years (41.5 months) after first surgery. Its expression was a negative independent prognostic marker for recurrent-free survival and overall survival. Furthermore, endothelial IDO expression was associated with reduced CD8^+^ T-cells and increased FoxP3^+^ Tregs in the tumor microenvironment. Interestingly, in patients with IDO^+^ ECs in the sentinel node enhanced IDO expression in the peripheral blood was detected, suggesting that endothelial IDO expression impacts systemic immunity (86). Similar observations were made in colorectal cancer, including a highly consistent expression pattern in the primary tumor, TDLNs (both tumor-invaded and tumor-uninvaded) and distant metastases (87). Endothelial IDO expression was more prevalent in MSI-H tumors compared to microsatellite stable (MSS) tumors. A negative effect on recurrence-free survival was observed for endothelial IDO expression, independent from disease stage, MMR status and CD8 count in the primary tumor.

In contrast, low IDO mRNA in the primary tumor of renal cell cancer patients was an independent unfavorable prognostic marker (88). Immunohistochemical analyses revealed that IDO was exclusively expressed by ECs, in contrast to the tumor cells which were IDO-negative. Another study in renal cell carcinoma confirmed absence of tumoral IDO expression and revealed that responders to anti-PD-1 therapy had stronger endothelial IDO expression compared to non-responders (89).

Little is known on the signaling pathways inducing IDO expression in ECs. Human umbilical vein ECs were reported to induce IDO upon stimulation with IFNγ (82) or TNFα, and these act synergistically when combined (81). IFNγ was also evidenced to upregulate IDO expression in human saphenous endothelial cells (164) and human corneal endothelial cells (165). In rats, IDO expression was induced in ECs by IFNγ-mediated activation of IKKα, which in turn stimulates the non-canonical NFκB pathway (83). In human invasive ductal carcinoma, IFNγ was demonstrated to be a potent inducer of endothelial IDO expression, which subsequently negatively affected the synthesis and secretion of stromal thrombospondin 1 (TSP1) via Trp deprivation. Reduced expression of TSP1, which is a large matricellular glycoprotein, supports cancer cells to evade tumor dormancy (90). Intriguingly, IDO was also induced when ECs were co-cultured with a tumorigenic metastatic triple negative breast cancer cell line (MDA-MB231). Tumor cells were a source of IFNγ in the co-culture, inducing IDO expression in ECs. Similarly, mRNA expression of IDO in lymphatic EC was significantly upregulated when co-cultured with CD4^+^ T-lymphocytes and a gastric cancer cell line (OCUM12) (162). Treatment of murine experimental melanoma with CD40 immunotherapy resulted in upregulation of IFNγ signaling and subsequent expression of IDO by ECs (84). Interestingly, CD40 mAb combined with an IDO inhibitor (epacadostat) delayed tumor growth in these mice, while activation of TILs was increased.

Lymphatic endothelial cells (LECs) have been implicated to attribute to a climate of systemic peripheral tolerance. Lymphatic vessels transport antigens and DCs to lymph nodes, where naïve cells are primed via cross-reaction. Despite their facilitating role in the migration and homeostasis of naïve T-cells, it has been described that LECs are involved in the induction of anergy of activated T-cells. An *in vitro* study observed that human LECs in lymph nodes induced IDO expression upon IFNγ-stimulation and impaired CD4^+^ T-cell proliferation when co-cultured (91). It is hypothesized that LECs promote CD8^+^ T-cell tolerance by the upregulation of inhibitory molecules including PD-L1 and IDO (166–168).

### Stromal Fibroblasts and Mesenchymal Cells

Tumor-surrounding stroma consists of fibroblasts, mesenchymal stromal cells, inflammatory cells, endothelial cells, and pericytes, which are all embedded in the extracellular matrix produced by fibroblasts (169, 170). Cancer associated-fibroblasts (CAFs) are the dominant stromal cell type and promote an immunosuppressive tumor microenvironment and tumor growth. IDO expression by CAFs was reported to be increased in the stroma of human esophageal cancers compared to non-tumor esophageal tissues (163). CAFs isolated from human metastatic melanoma and hepatocellular carcinomas have been documented to interfere with NK-cell mediated cancer cell killing (98, 99). CAFs expressed IDO and PGE2 when co-cultured with NK-cells, and impaired NK-cell secretion of granzyme B and perforin. Moreover, expression of NK-cell activation receptors such as NKp30 and NKp44 was downregulated. In addition to its suppressive effects on NK-cells, IFNγ-stimulated expression of IDO by dermal fibroblasts has been observed to induce apoptosis in CD4^+^ and CD8^+^ T-cells, B-cells and monocytes (100). In contrast to immune cells in the Trp-depleted microenvironment in this study, keratinocytes and endothelial cells were resistant and their proliferation was not altered. Another study highlighted low survival of the overall CD4^+^ T-cell population when co-cultured with IDO^+^ (compared to IDO^−^) fibroblasts (103). However, the frequency of the CD25^+^ FoxP3^+^ CD4^+^ subset was increased and these T-cells exhibited classic functional characteristics of Tregs (CTLA-4, IL10, and TGFβ). IFNγ is recognized as a potent inducer of IDO in fibroblasts (92, 93, 103). Additionally, the COX2/PGE2 pathway was suggested to mediate IDO induction in CAFs. Overexpression of COX2 by breast cancer cells was documented to trigger PGE2 secretion, subsequently upregulating STAT3-mediated transcription of IDO in fibroblasts (97). Strong expression of IDO by CAFs was associated with decreased disease-free and metastasis-free survival in breast cancer patients.

In addition to CAFs, mesenchymal cells are able to express IDO in the tumor-surrounding stroma. Bone marrow-derived mesenchymal stem cells [also known as multipotent mesenchymal stromal cells (171), both abbreviated MSCs] are recruited to sites of tissue injury, where they have the potential to differentiate into osteoblasts, adipocytes, and chondrocytes and mediate tissue repair (172). In cancer, bone marrow-derived mesenchymal stem cells are recruited to the primary tumor where they differentiate into CAFs (173–175). Similar to CAFs, IDO can be induced in mesenchymal stem cells by IFNγ (94–96). IDO expressing mesenchymal stem cells are also involved in inhibition of T-cell function (96) and expansion of Tregs (104). Mesenchymal stromal cells were demonstrated to be involved in the differentiation of monocytes into immunosuppressive M2-macrophages (101).

Besides CAFs and MSCs, the tumor-surrounding stroma also consists of pericytes. In normal conditions, pericytes participate in the regulation of blood flow and vessel permeability, and provide important mechanical and physiological support to ECs (176–178). The reciprocal communication between ECs and pericytes is crucial for vessel remodeling, maturation, and stabilization (177, 179, 180). Pericytes promote tumor angiogenesis, and once detached from tumor vessels they are able to differentiate into CAFs, thereby mediating an immunosuppressive tumor microenvironment (181). Intriguingly, resting pericytes were reported to activate alloreactive T-cells while IFNγ-stimulated pericytes suppress T-cell proliferation. Immunophenotyping of IFNγ-stimulated pericytes revealed IDO as one of the most upregulated gene transcripts, together with other inhibitory molecules such as PD-L1, PD-L2, and CAECAM1 (82). In this study, IDO expression in pericytes was verified as the principal mechanism accounting for negative regulation of T-cell proliferation. In primary ovarian cancer, IDO-positive tumor-associated vessels were predominantly mature blood vessels covered by pericytes (102).

## IDO in the Peripheral Blood

IDO expression in the peripheral blood can be measured by direct methods such as single-cell RNA sequencing and flow cytometry allowing intracellular detection of IDO in specific PBMC subsets. Another method is quantification of Trp and Kyn in plasma/serum via ultra-performance liquid chromatography–tandem mass spectrometry (UPLC-MS/MS). Since enzymatic activity of IDO is involved in the first and rate-limiting step of the catabolism of Trp to Kyn and its downstream metabolites, increased Kyn/Trp is regarded as a surrogate for enhanced IDO activity. Several studies in different solid and hematological cancer types have related increased serum (or plasma) Kyn/Trp ratio to worse survival outcome (4, 182–186). A higher Kyn/Trp ratio has been linked to metastasis, higher tumor size, and advanced disease stages (4, 187, 188). Furthermore, a role for serum Kyn/Trp in predicting resistance to systemic treatment has been reported in several malignancies (79, 187, 189–192). In a large number of stage IV melanoma and renal cell cancer patients treated with anti-PD-1 therapy, a high increase in Kyn/Trp during therapy compared to baseline was associated with significantly reduced progression-free survival (193).

Although Kyn/Trp seems to have clinical relevance, the exact source of this IDO expression is unclear since its detection in serum/plasma is an indirect method measuring enzymatic IDO activity. Serum Kyn/Trp in human penile squamous cell carcinoma patients correlated with IDO expression in cancer cells but not with IDO expression on tumor-infiltrating immune cells (194). A recent study profiling Kyn/Trp in more than 900 human cancer cell lines demonstrated that secreted Kyn can be attributed to both IDO and TDO expression by tumor cells (195). However, another study observed a correlation of the Kyn/Trp ratio with PD-L1 and IDO but not with TDO mRNA levels in melanoma samples after 4 cycles of anti-PD-1 immunotherapy (193). Nevertheless, the authors argued that additional sources of Trp to Kyn degradation outside the tumor may exist. In support of this, ovarian cancer patients with high serum Kyn/Trp had strong IDO expression in both tumor cells and pericytes (102). In glioblastoma, diminished therapeutic response to CTLA-4/PD-L1 mAbs in IDO^−/−^ mice compared to WT mice was observed, indicating the requirement for germline IDO to achieve maximal survival benefit from immune checkpoint therapy against brain tumors (196). Serum Kyn/Trp levels were significantly lower in IDO^−/−^ mice compared to WT mice. Notably, no change in Kyn/Trp levels of isolated brain from glioblastoma WT mice and IDO^−/−^ mice was noted. These findings suggest that non-tumor cell IDO activity contributes to a pool of Kyn in serum that facilitates responsiveness to immune checkpoint blockade. In line with these observations, serum Kyn/Trp measured by UPLC-MS/MS correlated with IDO expression in PBMCs measured by flow cytometric analysis of peripheral blood samples of melanoma patients (86). This correlation suggests that serum Kyn/Trp reflects metabolic activity of IDO-expressing circulating immune cells.

Only a few studies report on *in vivo* expression of IDO by specific subsets of immune cells in the peripheral blood. Munn et al. (197) observed low to undetectable levels of IDO in monocytes isolated from fresh PBMCs. Monocyte-derived CD123^+^ macrophages upregulated IDO expression when stimulated with IFNγ *in vitro*. Surprisingly, IDO was constitutively expressed in human CD123^+^ DCs in peripheral blood, but activation with IFNγ was still required for functional enzymatic activity. IDO^+^ CD123^+^ DCs expressed MHC II and costimulatory molecules and were effective stimulators of T-cell proliferation when incubated with an IDO-inhibitor, suggesting that these cells could act as competent APCs.

DCs are observed to constitutively express IDO, but an additional set of triggering signals during antigen-presentation is required for its activity. Monocyte-derived DCs obtained from peripheral blood of melanoma patients were demonstrated to upregulate IDO expression upon *in vitro* activation by CD40L and IFNγ. During an immune response, activated DCs interact with IFNγ-expressing CD8^+^ T-cells via CD40-CD40L ligation, subsequently mediating NFκB-dependent IDO upregulation in DCs (108, 198). Furthermore, IDO activity during DC activation was observed to be involved in the maturation of DCs, as Trp deprivation regulated expression of CCR5 and CXCR4 and DC responsiveness to chemokines (199). Another IDO-triggering signal can be elicited by DC B7-1/B7-2 ligation with CTLA-4/CD28, the latter being predominantly expressed by Tregs (200). Subsequent to the activation of IDO on DCs by activated Tregs, IDO^+^ DCs drive differentiation of naïve CD4^+^ CD25^−^ T-cells into mature Tregs (201–204).

Expression of IDO by DCs has been demonstrated in peripheral blood of melanoma patients. IDO expression was predominantly found in CD123^+^ pDCs in addition to monocytic MDSCs (mMDSCs), and to a lesser extent in polymorphonuclear MDSCs (pmnMDSCs) (86). Circulating pDCs and MDSCs were key players in the systemic response in melanoma, as they had an independent prognostic effect on survival of melanoma patients (205). Furthermore, IDO activity in peripheral blood was positively correlated with levels of circulating PD-L1^+^ CD8^+^ T-cells and CTLA-4^+^ Tregs, underlining the close interconnection between these immunosuppressive markers in the blood circulation (86, 206). In early epithelial ovarian cancer, the level of IDO^+^ mMDSCs and IDO^+^ pmnMDSCs was significantly higher in the peripheral blood compared to the tumor microenvironment (207).

## Discussion

IDO expression in cancer has been described in a wide variety of cells both at the level of the tumor microenvironment and the peripheral blood. Depending on the exact location of expression, different induction pathways and effector functions have been observed. Several inflammatory cytokines such as IFNγ, IL-1, IL-6, IL-32, TNFα, and TGFβ were evidenced to drive IDO induction. Soluble factors excreted by tumor cells such as Wnt5a and sTGFBR3 are capable of inducing IDO in immune cells. Vice versa, immune cells such as CD8^+^ T-cells in highly inflamed tumors were found to mediate induction of IDO in tumor cells via IFNγ signaling. The mechanisms that induce IDO expression and its various physiological and pathophysiological roles are currently incompletely understood but may be important in human biology in general and medical oncology in specific.

There is ample evidence on the role of IDO in tumor immune escape. The suppressive effects on T-cell responses in the different compartments of the tumor microenvironment are well-documented in both animal and human studies. Enzymatic activity of IDO in tumor cells, as well as in endothelial cells, APCs, MDSCs, and fibroblasts has been reported to stimulate anergy of effector T-cells, while Treg activity is enhanced. In addition, naïve CD25^−^ CD4^+^ T-cells are polarized toward the immunosuppressive FoxP3^+^ CD25^−^ CD4^+^ phenotype while their conversion into Th17-like T-cells is blocked. In addition to the inhibition of antitumor immune responses, tumoral IDO expression promotes lymphangiogenesis and neovascularization, further facilitating tumor progression.

In the different compartments, IDO expression is closely interconnected with other immune checkpoint molecules already targeted by current immunotherapies. In the local tumor microenvironment, CTLA-4 expression in Tregs upregulates IDO in DCs, which reciprocally promotes Treg activation. This interplay of immune checkpoints is also evidenced in the peripheral blood, where IDO expression by PBMCs was demonstrated to be associated with increased circulating PD-L1^+^ CD8^+^ T-cells and CTLA-4^+^ Tregs. In addition, blockade of CTLA-4 and/or PD-1 has been reported to upregulate IDO expression as a result of the increased IFNγ-production by reactivated effector T-cells. Immunomonitoring of blood samples can highlight such dynamic shifts in ongoing immune responses. In NSCLC, RCC and melanoma patients the baseline value of systemic Kyn/Trp as well as its dynamics during treatment course were associated with patient outcome (189, 193). In this way the Kyn/Trp ratio could be a marker best capturing IDO activity at a specific moment and perhaps could have relevance in therapeutic monitoring.

In the context of immunotherapy, the immunosuppressive role of host cell expressed IDO is supported by a striking delay in tumor growth in anti-CTLA-4 treated IDO knockout mice compared to WT mice (208). Pharmacological inhibition of IDO by 1-methyl-tryptophan (1MT) combined with anti-CTLA-4 resulted in rejection of established tumors and resistance to secondary challenge in mice inoculated with B16 melanoma. Tumor rejection by anti-CTLA-4/1MT therapy was associated with enhanced infiltration of functional CD8^+^ and CD4^+^ T-cells in the tumor. Notably, the combination therapy is synergistic irrespective of detectable IDO expression in tumor cells, though therapeutic efficacy was reduced against B16 melanoma cells engineered to overexpress IDO. Synergistic retardation of tumor outgrowth by anti-CTLA-4, anti-PD-L1 and/or IDO inhibition (INCB23843) was confirmed in a murine B16.SIY melanoma model (209). In preclinical models, IDO blockade has been demonstrated to be effective as part of combination therapy including immune checkpoint therapy, DNA-damaging chemotherapy and radiotherapy (21, 210). In a 4T1 breast tumor bearing mouse model, local radiotherapy combined with intratumoral CpG upregulated IDO expression in neoplastic epithelial cells. Systemic 1MT significantly decreased IDO activity (as measured by serum Kyn/Trp) and augmented the antitumor efficacy of local radiotherapy and intratumoral CpG (210).

Several IDO inhibitors tested in phase 1/2 clinical trials showed promising results. INCB024360 (epacadostat), a competitive, selective inhibitor of IDO, was well-tolerated in a first-in-human phase 1 study with near maximal inhibition achieved (measured by decreases in plasma Kyn levels) at doses ≥100 mg twice daily (BID) (211). Phase 1/2 studies evaluated epacadostat in combination with anti-CTLA-4 (ipilimumab) (212) and anti-PD-1 [nivolumab (213) and pembrolizumab (214)] and showed encouraging antitumor activity in multiple advanced solid tumors. However, a phase 3 trial in unresectable or metastatic melanoma (ECHO-301/KEYNOTE-252) failed to show any benefit of the addition of epacadostat to pembrolizumab (215). Results of this trial raised questions concerning IDO inhibition strategies in cancer treatment, however there are certain caveats (216). The dose of epacadostat used in ECHO-301 is debated as a maximum reduction in Kyn levels of only 50% was seen for this 100 mg dose in phase 1 studies. In addition, pharmacodynamic data reported for epacadostat were based on plasma measurements of Kyn, while IDO expression in the tumor microenvironment was not investigated (211). Baseline IDO expression or Kyn/Trp levels were not employed as inclusion criteria in the ECHO-301 study, and patients who previously received an adjuvant CTLA-4-inhibitor or interferon treatment were also included. Importantly, in a melanoma cohort receiving adjuvant IFN-α2b enhanced Kyn/Trp levels were detected compared to untreated patients (217). Furthermore, melanoma patients who did not respond to anti-CTLA-4 (ipilimumab) combined with stereotactic body radiotherapy showed an increase in the Kyn/Trp ratio during treatment compared to baseline Kyn/Trp (191). These data indicate that certain (immuno-) therapies may upregulate IDO activity, raising the question whether an enhanced dose of epacadostat would have been needed in the ECHO-301 study in order to fully block IDO activity.

BMS-986205, an irreversible IDO1 inhibitor, was demonstrated to reduce both serum (>60% mean reduction at a dose from 100 to 200 mg) and intratumoral (up to 90% reduction) Kyn levels (218). Currently, BMS-986205 in combination with anti-PD-1 therapy is investigated in several phase 2 trials (219–221). Besides IDO-specific inhibitors, other approaches to inhibit this pathway continue to be considered. Indoximod, a Trp mimetic, restores the activity of master metabolic kinase mTORC1 in effector T-cells, reversing autophagy triggered by Trp depletion (222). By targeting a downstream convergent effector mechanism used by IDO, as well as IDO2 and TDO, indoximod might prove less sensitive to negative feedback mechanisms that may result in treatment resistance (223).

Further research is needed to better understand the exact biological functions of IDO but also of the two other Trp-degrading enzymes IDO2 and TDO in the different compartments in cancer (224, 225). Increased insights in how these enzymes affect cancer immune escape and disease outcome could facilitate patient stratification in future clinical studies. The next step would be to investigate how these insights can be used to reverse this negative immune climate, thereby paving the way to personalized immuno-oncology possibly already in an early stage of cancer.

## Author Contributions

All authors had a substantial contribution to the manuscript, and approved the submitted version. AM and LB wrote the manuscript. AM generated the figure and MD assisted in the revision and made the table.

## Conflict of Interest

LB was participant in advisory board Incyte, München, Germany, June 2017 and gave an internal training for Incyte European division, Amsterdam, October 2017. The remaining authors declare that the research was conducted in the absence of any commercial or financial relationships that could be construed as a potential conflict of interest.
